# Draft genome sequences of three *Pantoea* strains promoting the growth of wheat, isolated from desert plant rhizosphere soils

**DOI:** 10.1128/mra.00474-25

**Published:** 2026-02-11

**Authors:** Yungang Liang, Fangfang Xu, Kai Tang, Fuying Feng, Huirong Liu

**Affiliations:** 1 Laboratory for Environmental Microbiology and Biotechnology in Arid and Cold Regions, College of Life Sciences, Inner Mongolia Agricultural University 117454 , Hohhot, China; 2 Inner Mongolia Key Laboratory of Biomanufacturing Technology, Hohhot, China; 3 PLA Army Chemical Defense College 659718 , Beijing, China; The University of Arizona, Tucson, Arizona, USA

**Keywords:** *Pantoea*, plant growth-promoting rhizobacteria, genome sequencing

## Abstract

We report three draft genome sequences of wheat growth-promoting *Pantoea* spp. isolated from desert plant rhizosphere soils in western Inner Mongolia, China. Genomes were sequenced using Illumina NovaSeq 6000, yielding an average genome size of 4,285,493 bp and a mean GC content of 53.4%.

## ANNOUNCEMENT

**Fig 1:**
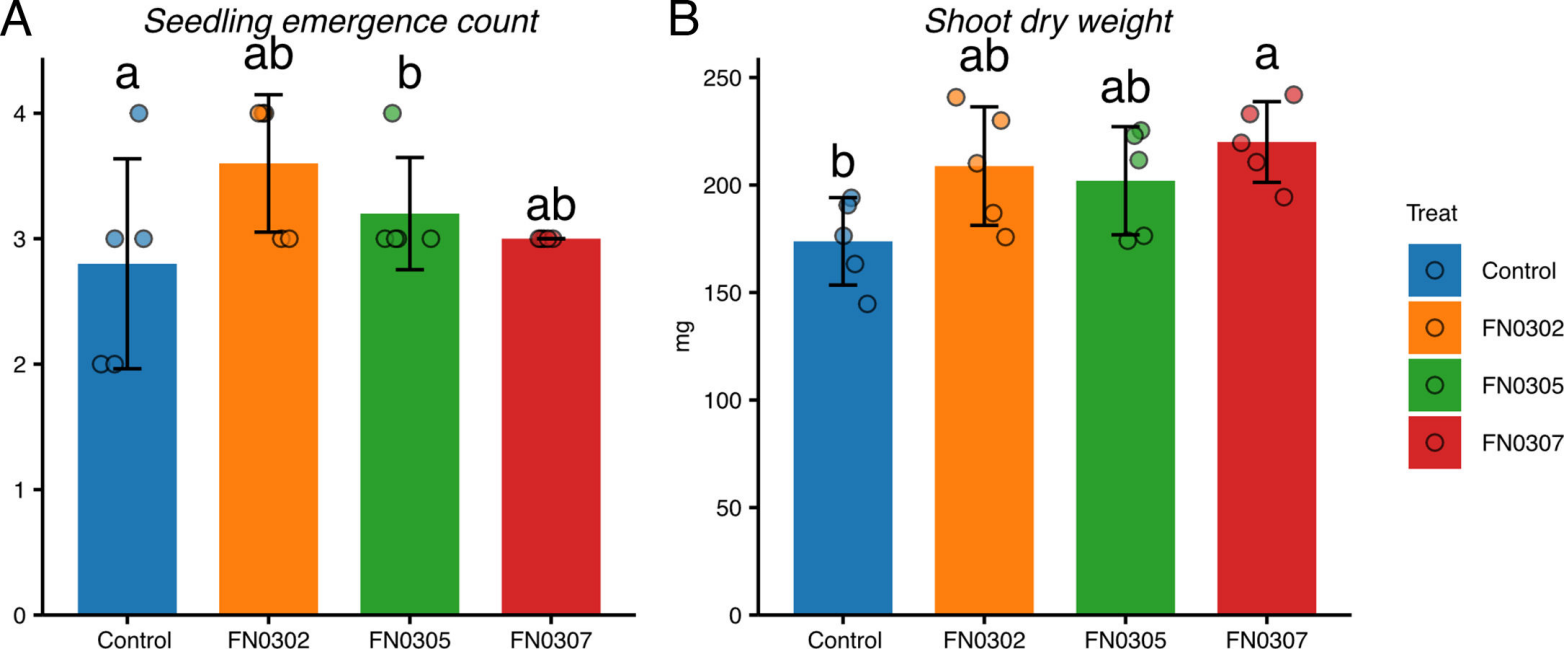
Effects of three *Pantoea* strains on wheat seedling growth. (**A**) Seedling emergence count and (**B**) shoot dry weight of wheat plants inoculated with strains FN0302, FN0305, and FN0307 compared with the uninoculated control. Bars represent mean values, error bars indicate standard deviation, and points represent individual replicates. Different letters above bars indicate significant differences among treatments (*P* < 0.05).

*Pantoea* spp. are recognized as potential plant growth-promoting rhizobacteria (1). To better understand their potential to enhance plant growth, we isolated and sequenced three *Pantoea* strains from desert plants. Rhizosphere soil samples, defined as soil adhering to the roots after shaking off bulk soil, were collected from desert plants at Bayannur, Inner Mongolia, China (40°14’28”N, 107°05’43”E) in October 2015. One gram of rhizosphere soil was serially diluted in 9 mL sterile saline, spread onto 1/2 Reasoner’s 2A agar, and incubated at 28°C for 72 hours. Single colonies were picked and purified through successive streaking. Strains FN0302 and FN0307 were isolated from *Caragana intermedia* Kuang et H. C. Fu rhizosphere soils, and strain FN0305 was isolated from *Ammopiptanthus mongolicus* (Maxim. ex Kom.) Cheng f. rhizosphere soil. All three strains promoted wheat seedling growth under salt stress in pot experiments. Each strain was cultured in 1/2 Reasoner’s 2A broth at 28°C for 72 h with shaking, centrifuged, and resuspended to an OD₆₀₀ of 1.0 in 0.1% (wt/vol) sterile CMC-Na solution for seed soaking (2). Treated seeds were sown in pots with soil salinized to 0.4% NaCl, with five seeds per pot and five replicates per treatment. Seedlings were sampled after 30 days for phenotypic measurements. Under these conditions, all three strains increased seedling emergence rates and shoot dry weights compared to uninoculated controls (Fig. 1).

Single colonies were inoculated into 1/2 Reasoner’s 2A broth and incubated at 28°C with shaking for approximately 24 hours until stationary phase, then harvested by centrifugation at 5,000 × *g* for 10 min at 4°C. Genomic DNA was extracted using the SDS method (3) and processed into sequencing libraries using the NEBNext UltraDNA Library Prep Kit for Illumina (NEB, USA) according to the manufacturer’s instructions. Whole genomes were sequenced using the Illumina NovaSeq 6000 platform at Novogene Bioinformatics Technology (Beijing, China), generating 150 bp paired-end (PE150) reads. Raw reads were processed using readfq software version 10 with parameters: “–rq1 input_1.fq, –rq2 input_2.fq, –oq1 out_1.fq, –oq2 out_2.fq, –adp1 adapter_1.lst, –adp2 adapter_2.lst, –Q QUAL,PERCENT, –C QUAL,PERCENT, –N PERCENT, –alen INT, –amis INT, –dup, –gz, –check1 read1.check, and –check2 read2.check.” Clean data were assembled using SOAPdenovo (4, 5) version 2.04, ABySS (6) version 1.3.6, and SPAdes (7) version 3.10.0. Assembly results were integrated using CISA (8) version 1.3, selecting the assembly with the fewest scaffolds. GapCloser (9) version 1.12 was used to fill assembly gaps, removing fragments below 500 bp.

The genome assemblies were deposited in NCBI GenBank, where they were annotated using the NCBI Prokaryotic Genome Annotation Pipeline (10) for public release. For downstream comparative and functional analyses described below, the genomes were re-annotated using Bakta (11) version 1.11.0. Genome sizes ranged from 4,204,445 to 4,345,745 bp with GC content from 53.34% to 53.48%. Contamination and completeness were assessed with CheckM (12) version 1.2.3. Average nucleotide identity (ANI) analysis using FastANI (13) version 1.32 indicated that all three strains were closely related to *Pantoea* alhagi LTYR-11Z (GenBank accession number CP019706). Sequencing and annotation details are provided in Table 1.

**TABLE 1:** Isolation details and genome assembly statistics of three *Pantoea* strains

	FN0302	FN0305	FN0307
Host plant	*Caragana intermedia* Kuang et H. C. Fu	*Ammopiptanthus mongolicus* (Maxim. ex Kom.) Cheng f.	*Caragana intermedia* Kuang et H. C. Fu
BioSample	SAMN48141140	SAMN48141141	SAMN48141142
Whole-genome sequencing (WGS) accession no.	JBNJSM000000000	JBNJSN000000000	JBNJSO000000000
SRA accession number	SRR33873240	SRR33873239	SRR33873238
DNA genome size (bp)	4,204,445	4,345,745	4,306,289
Illumina reads (read pairs)	4,068,272	1,642,470	2,000,000
Coverage	290.28	113.38	139.33
Assembly *N*_50_ value (bp)	2,260,127	439,991	625,342
Assembly *N*_90_ value (bp)	128,349	131,528	209,838
GC content (%)	53.5	53.3	53.4
No. of contigs	14	26	11
rRNA gene count	7	11	6
tmRNA gene count	1	1	1
tRNA gene count	75	78	74
Coding DNA sequence (CDS) count	3,840	3,947	3,976
Contamination (%)	0.26	0.26	0.46
Completeness (%)	99.79	99.79	97.82
Most closely related genome according to whole genome sequence in NCBI (% ANI)	*Pantoea alhagi* LTYR-11Z (96.24)	*Pantoea alhagi* LTYR-11Z (96.34)	*Pantoea alhagi* LTYR-11Z (96.26)

## Data Availability

This project has been deposited in DDBJ/ENA/GenBank under accession number PRJNA1255394. BioSample numbers, SRA accession numbers, and the whole-genome shotgun (WGS) accession numbers are detailed in Table 1.
